# Supplementary material for: Investigating Candida glabrata Urinary Tract Infections (UTIs) in Mice Using Bioluminescence Imaging
Source: J Fungi (Basel). 2021 Oct 9;7(10):844. doi: 10.3390/jof7100844 (PMC8538756; doi:10.3390/jof7100844)
Supplement: Supplementary file 1 [file jof-07-00844-s001.zip › jof-1400818-Supplementary.pdf]

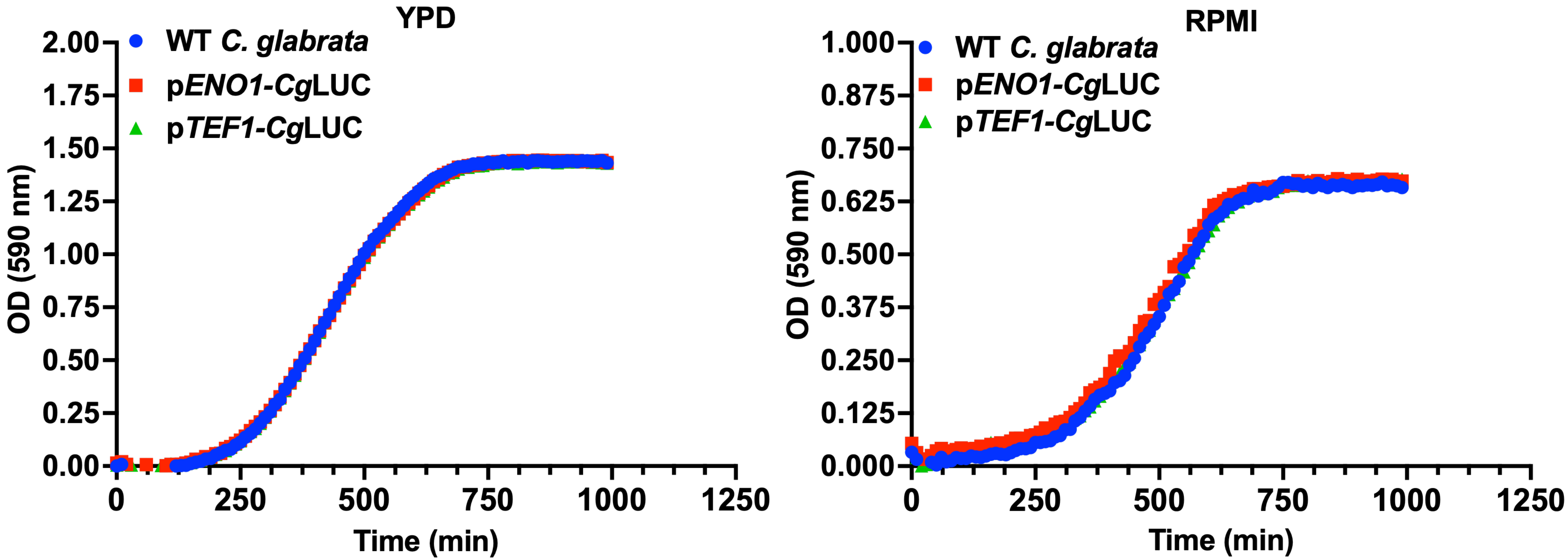

Figure S1: Luminescent *C. glabrata* strains have a similar growth rate compared to the wild type strain in YPD and RPMI (0.2% glucose).
